# Distinguishing Between AI-Generated and Human-Written Electronic Residency Application Service (ERAS) Personal Statements in Otolaryngology

**DOI:** 10.7759/cureus.99202

**Published:** 2025-12-14

**Authors:** Rahul Menon, Donald Solomon, Mia Berenson, Vlad Kushnir, Yekaterina Shapiro

**Affiliations:** 1 Medical School, Cooper Medical School of Rowan University, Camden, USA; 2 Otolaryngology - Head and Neck Surgery, Cooper University Hospital, Camden, USA; 3 Medical School - Graduate Medical Education, Rowan-Virtua School of Osteopathic Medicine, Stratford, USA; 4 Law, Cooper University Hospital, Camden, USA

**Keywords:** artificial intelligence, chatgpt, otolaryngology, personal statements, residency applications

## Abstract

Background

Generative AI tools, such as ChatGPT, raise concerns about authenticity and fairness in residency applications. Since the transition of USMLE Step 1 to pass/fail, residency programs have placed greater emphasis on qualitative components such as the personal statement. The integrity of this document is critical, as it informs interview invitations and final rank decisions. However, it remains unclear whether reviewers can reliably distinguish AI-generated from human-written content and how this may affect the residency selection process.

Objective

In this small pilot study, we evaluated the ability of generative AI, specifically ChatGPT 4.0 (OpenAI, Inc., San Francisco, CA, USA), to produce convincing personal statements for otolaryngology residency applications and explored whether a limited sample of expert reviewers could distinguish AI-generated from human-written content. We used 2019 applicant-written statements because they predated the widespread availability of generative AI, ensuring a true “human-only” comparison group.

Methods

In 2024, ChatGPT was given a sophisticated and detailed prompt to generate five otolaryngology residency personal statements. These were combined with five de-identified, applicant-written statements submitted in 2019. Four otolaryngologists from a single academic medical center and four attorneys, blinded to the origin of the statements, rated them using a standardized rubric for readability, originality, persuasiveness, and interview desirability. An AI detection tool (Scribbr, Amsterdam, The Netherlands) also analyzed each statement. Statistical analyses included paired t-tests and inter-rater reliability assessment.

Results

Eight blinded reviewers (four otolaryngologists and four attorneys) evaluated 10 personal statements (five AI-generated and five human-written). Mean ratings for readability (AI 3.63 ± 0.22 vs. human 3.53 ± 0.40), originality (3.65 ± 0.16 vs. 3.53 ± 0.39), persuasiveness (3.35 ± 0.76 vs. 3.43 ± 0.36), and interview desirability (3.40 ± 0.51 vs. 3.30 ± 0.58) showed no significant differences (all P > 0.05), but given the small sample size, the study was underpowered (post hoc estimated power 14.1% for medium effect size), limiting the ability to detect statistically significant differences. Inter-rater reliability was moderate (intraclass correlation coefficient = 0.66), indicating consistent scoring trends across reviewers. The AI detection tool correctly flagged 93.6% of ChatGPT-generated text and had an AI probability rating ranging from 0% to 20% with a mean of 4% in the human-written text. Reviewer profession (medical vs. legal) did not significantly influence scoring patterns. Although underpowered due to a small sample size, the findings suggest that ChatGPT-generated personal statements are comparable in quality and perceived authenticity to those written by applicants, yet remain largely detectable by current AI-detection algorithms.

Conclusions

These findings suggest that ChatGPT can generate statements that may be difficult to distinguish from applicant-written work, but AI detection tools remain effective in identifying synthetic text. For residency programs, these results highlight the need for clear guidelines on AI use, integration of detection strategies, and ongoing research to understand how AI may affect perceptions of authenticity in applicant evaluation.

## Introduction

Over the past few years, AI has opened paths to innovation and improvement in healthcare. The further we step into the era of AI, the more important it has become to explore how generative AI might reshape more traditional processes, particularly in its use and potential abuse by medical student applicants in the Electronic Residency Application Service (ERAS).

In spite of more quantitative metrics like USMLE STEP exam scores and medical school grades, the personal statement remains an important tool used by residency programs, with 74-78% of them using the statement in their interview selection process and 48-54% using it in their final rank of candidates [1]. Moreover, the USMLE STEP 1 Exam moving to a pass/fail format places much greater emphasis on other metrics, like the personal statement, when evaluating applicants [2]. In 2021, the otolaryngology match saw over 550 medical students apply for one of only 350 otolaryngology residency spots, which translates to a 63% match rate for the year [3]. For the budding applicant, a poignant and effective personal statement may make the difference between a successful and an unsuccessful residency match. While most personal statements draw on human experiences, the uniqueness of those experiences is sometimes called into question. One analysis of applicants to internal medicine, family medicine, and general surgery showed that 97% of applicants used an opener that included some combination of either a personal narrative and/or reasoning on pursuing medicine or the specialty of choice. A different analysis of anesthesia applicants’ personal statements cited the discussion of a significant patient case or the illness of a family member or friend as being more original [1]. While each of these experiences can be written in a manner unique to each applicant, it is evident that successful residency application personal statements typically have a combination of these elements.

AI algorithms make use of artificial neural networks that are essentially trying their best to mimic the human brain and its own interconnected network of neurons. As computational power has increased greatly over the years, so too has the depth of the neural networks, allowing them to better mimic the human brain and assume greater functionality [4]. In the healthcare setting, generative AI has shown promise in the realm of documentation automation, boasting the ability to write operative and consultation reports as well as consent forms [5]. While some are skeptical of the quality of AI-generated writing, one study demonstrated that 32% of AI-generated abstracts were falsely graded by blinded human reviewers as human work, and reviewers cited the surprising difficulty of distinguishing between the two [6]. With regard to personal statement writing, one article had a group of 10 plastic surgery residents and faculty members rate, in a blinded fashion, original personal statements and those generated by AI for things such as readability, originality, syntax, and whether an interview offer should be extended. Interestingly, there were no significant differences between the ratings of the AI-generated pieces and the applicant statements [7]. Even though there was not a significant difference, it was found that the raters were less likely to extend an interview when essays were AI-generated, citing a 58% chance of extending an interview for the AI versus 78% for the human applicant. However, one limitation gleaned from this study was the brevity of the prompt that was given to ChatGPT. Moreover, new iterations of the generative AI model have come out since then. Regardless, the authors of the article posit that the current application process requires reevaluation; for example, targeted questions and prompts may add more value in assessing an applicant’s critical thinking skills and writing ability.

In another study, ChatGPT was used to craft personal statements that were then sent to anesthesia residency programs along with the original work of applicants. These personal statements centered around the prompt of including common applicant experiences of being involved in athletics or cooking, while also including meaningful patient stories. The study found that more than 90% of anesthesia program directors found the AI-generated statements acceptable, and 80% reported that they were unable to distinguish which statements were AI-generated [8]. Notably, the prompt given to the algorithm had a great influence on the finished product that ChatGPT was able to produce. If this tool is used in a particular way, ChatGPT can create personal statements that many program directors find acceptable and very difficult to distinguish from unique, applicant-written ones. Early reports suggest that AI is already influencing medical education and scholarly writing, raising concerns about authorship integrity and the role of technology in trainee evaluation [9,10]. While it is unlikely that many applicants will rely solely on fully AI-generated personal statements, the potential for hybrid use (AI-assisted editing, restructuring, or polishing) is high. This complicates the boundary between authentic self-expression and algorithmically enhanced writing.

The goal of this pilot study is to evaluate whether generative AI, specifically ChatGPT-4, can produce specialty-specific, otolaryngology residency application personal statements that are indistinguishable from those written by human applicants after using detailed prompting. We further aim to compare the ability of domain experts (otolaryngologists) and nonmedical specialist professionals (attorneys) and an AI detection tool to differentiate between these AI- and human-written essays in an effort to inform discussions surrounding the ethics of AI usage, personal statement authenticity, and current evaluation standards of the traditional residency application process. Distinguishing between AI- and human-written content is not simply an academic exercise; it carries implications for fairness in residency selection, the authenticity of applicant narratives, and the trust program directors place in application materials.

## Materials and methods

Study aim

This study aimed to assess whether expert raters could distinguish AI-generated from human-written otolaryngology personal statements and to evaluate whether detection tools corroborate these assessments.

Study design

The aim of this study is to assess the ability of ChatGPT 4.0 (OpenAI, Inc., San Francisco, CA, USA) in generating personal statements for otolaryngology residency applications, simulating the prompting that a medical student may utilize to fully generate an AI essay. In May 2024, ChatGPT 4.0 was prompted to draft five personal statements for otolaryngology residency. With regard to prompt engineering, specificity, structure, and context-rich guidance in a prompt significantly influence AI output quality, ultimately yielding a tailored, coherent, and narratively rich product that aligns with professional expectations. For the prompting, we instructed the AI to generate a compelling, personalized residency personal statement for otolaryngology, focusing on integrating personal life experiences, including discussion on unique and meaningful hobbies, incorporating vividly detailed or fabricated yet believable patient interactions and research experiences, emphasizing why the applicant would be a strong fit, and producing a finished product that is 600-850 words. Central to the prompting was the goal to sound as human as possible. Word counts were not formally standardized; however, prompts were designed to yield statements of typical ERAS length (approximately 600-850 words). This variability is acknowledged as a study limitation.

Human-written personal statements for otolaryngology were collected with permission and de-identified from applicants who applied to the Otolaryngology Residency Program at our institution in 2019. In the final sample, 10 statements were included: five AI-generated and five randomly selected using a simple random number generator from the pool of de-identified 2019 statements available at our institution. Given the small sample size of statements and reviewers, the study was underpowered with a post hoc estimated power of 14.1% for a medium effect size, limiting the overall ability to detect statistically significant differences.

These statements were compiled into a survey, and the statements were graded on a rubric for originality, readability, how convincing the essay is, and the desire to extend an interview to the writer of the personal statement. The exact prompt entered into ChatGPT and the rubric used for evaluating the essays can be found in the Appendices section. The rubric (readability, originality, persuasiveness, and interview desirability) was adapted from prior work on AI-authored abstracts and residency statements [7,8] and modified by the authors for face validity. Each query for a new AI-generated personal statement was executed in its own ChatGPT session rather than in sequence to avoid any bias. The otolaryngologists included both faculty involved in applicant selection and clinical educators. Attorneys were chosen as a comparator group because of their professional expertise in written advocacy and persuasive communication, though we acknowledge they are not typically part of residency admissions processes.

Data collection and analysis

The AI-generated essays and human-written statements were individually assessed by four otolaryngologists from our institution, blinded to the source of each statement. Based on a rubric provided to reviewers, the statements were graded for originality, readability, how convincing the essay is, and the desire to meet the writer of the personal statement on a subjective range (Likert scale from 1 to 5). Four attorneys were also surveyed to comment and check if they could differentiate between the AI-generated and human-written statements. Attorneys were chosen as a comparison group due to their expertise in written communication, argumentation, and persuasive writing, making them a reasonable control for assessing how domain-specific experience influences detection accuracy. Finally, the personal statements were passed through an AI detection tool (Scribbr, Amsterdam, The Netherlands) to check their performance against our human reviewers.

The evaluations of each essay were used to generate descriptive statistics regarding the scoring categories for either type of personal statement. The scoring for the AI-generated essays and human-written essays was then compared using a paired t-test. Paired t-tests were chosen to compare AI vs. human ratings because the primary outcome was mean score differences. Correlation coefficients (Pearson/Spearman) were not used, as the objective was not to measure associations between raters but to compare overall group means. This data was also stratified by profession to see if that affected reviewer ability. Statistical significance was set at P < 0.05. To evaluate consistency in grading between reviewers, inter-rater reliability was assessed using the intraclass correlation coefficient for each type of personal statement. An online AI detection tool was assessed by computing the mean of the reported percentage of suspected AI-generated text in the AI-written personal statement group versus the human-written one. All statistical analyses were conducted using R (Version 4.4.2).

IRB statement

This study was reviewed and deemed exempt by the Cooper University Health Care Institutional Review Board.

## Results

**Figure 1:**
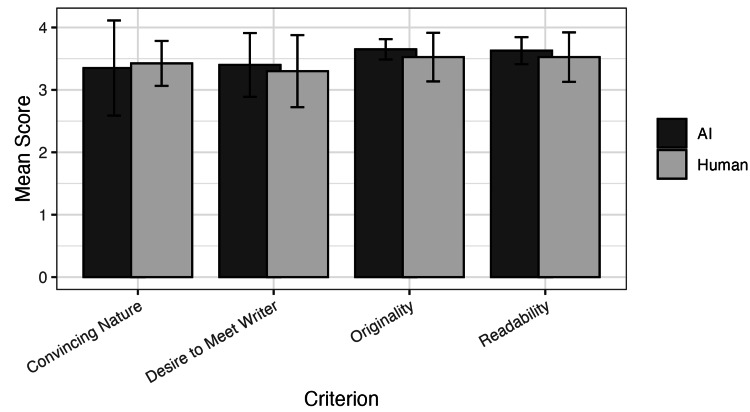
Mean scores by criterion for AI vs. human personal statements Mean reviewer scores for each evaluation criterion. Error bars represent standard deviations, indicating variability in ratings.

Eight survey members (four otolaryngologists and four attorneys) reviewed 10 personal statements regarding the readability, originality, and persuasiveness of the essays, along with a measure of whether the reviewer would like to interview the writer. Between the human and AI-generated personal statements, the means are similar, with the highest variance being in how convincing the AI-generated personal statements were to the reviewer (Figure 1). Ultimately, however, there was no significant difference (all P > 0.05) in these criteria between the AI-generated and human-written personal statements (Table 1). The intraclass correlation coefficient was 0.66 for both AI and human-written statements, indicating moderate inter-rater reliability. Given the small sample size, the study was underpowered (post hoc estimated power 14.1% for a medium effect size), limiting the ability to detect statistically significant differences.

**Table 1:** Paired t-test comparing AI vs. human criterion scores

Criterion	T-statistic	P-value
Readability	-0.32638	0.745
Originality	0.54167	0.5896
Convincing nature	-0.2839	0.7773
Desire to meet writer	0.36992	0.7125

**Figure 2:**
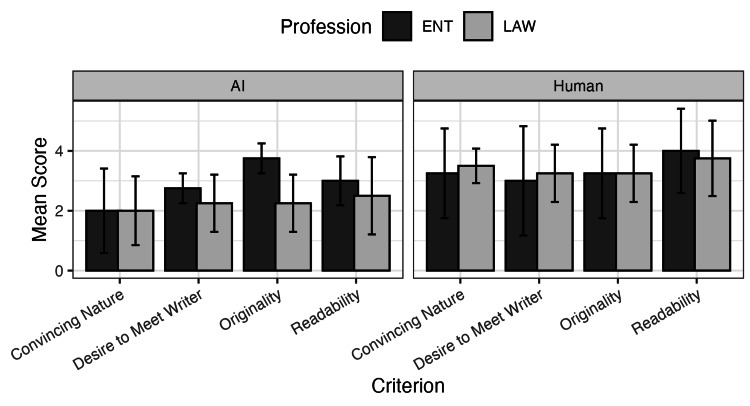
Mean scores by profession and statement type for each criterion Reviewer scores stratified by profession and statement type. Error bars represent standard deviations, reflecting rater variability across evaluation criteria.

When stratifying the data by the profession of the reviewer, this revealed no statistically significant difference between attorneys and otolaryngologists regarding the assessed personal statement criteria (Figure 2). This holds true even when stratifying by evaluations done for the two different personal statement types, human-written and AI-generated.

In determining whether a personal statement was the product of AI or not, the AI detection tool detected an average of 93.6% (82-100%) AI-generated text in the personal statements that were the full product of AI, as well as an average of 4% (0-20%) of AI-generated text in the human-written personal statements.

## Discussion

In this small pilot study examining AI-generated ERAS personal statements to applicant-written ones, no statistically significant difference in readability, originality, and persuasiveness was detected between them. AI-generated personal statements, on paper, may appear similar to applicant-written statements in readability, originality, and persuasiveness, and generative AI seems to have capabilities in creating and synthesizing language in a way that mimics human writing.

Prior studies in other specialties echo these findings. Patel et al. (2023) in Plastic Surgery and Johnstone et al. (2023) in Anesthesiology both observed that reviewers rated AI-generated statements similarly to authentic ones [7,8]. In the recent study by Steele et al., five AI-generated and five authentic ERAS personal statements for emergency medicine were blinded and evaluated by 30 members of the Council of Residency Directors for writing quality, expression of personal attributes, and influence on interview decisions. Similarly to our study, the authors found no statistically significant difference between AI-generated and human-written statements across grammar and writing style, personal attribute expression, overall quality, or perceived influence on interview decisions [9]. This suggests that, at least in an emergency medicine context, AI-generated statements can achieve parity with human ones from the perspective of reviewers. Our study, which also focused on specialty-specific reviews from otolaryngologists, had moderate inter-rater reliability (intraclass correlation coefficient = 0.66), aligns with these other reports, and underscores subjective variability in detecting synthetic text. Unlike those studies, our inclusion of nonmedical raters (attorneys) provides insight into how domain expertise affects detection accuracy. Together, these findings indicate a cross-disciplinary challenge in distinguishing AI authorship and emphasize the need for structured guidelines and reviewer training.

With regard to AI detection, one study found that in personal statements for medical school admission, a process not too dissimilar to the residency application process, AI-generated essays were rated higher and were indistinguishable from human-written essays by application readers [10]. Moreover, this same study found that while detection tools, in this case ZeroGPT, boasted a high accuracy (91%), they still had a high rate of false positives [10]. Another study evaluated a different AI detection tool, GPTZero, which was also able to identify AI-generated ERAS statements with high accuracy (91.7%) but also reported a modest AI probability score of 11% in the human-written essays [11]. In one study by Cumbo et al., 25 writing samples were analyzed with three AI-detector tools (GPTZero, Undetectable AI, and Winston AI). The results indicated high detection rates for AI-generated statements but also significant variability and risk of false positives when applied to human-written personal statements (detection rates ranging from 3% to 100% of content flagged as AI) [12]. These findings at large align with our study’s findings, which also showed a high accuracy rate for the AI detection tool, though in one purely human-written essay, it still falsely identified up to 20% of the content as AI-generated. Overall, these findings demonstrate the improved AI-detection ability of these tools when compared to human reviewers, but caution should still be advised when implementing these tools into standardized review processes due to the false positive rates.

When it pertains to the input of generative AI on academic writing, many publishers and journals are still formulating policies; many are requesting researchers to disclose if ChatGPT has been used in the creation of articles [13]. Notably, some research articles have already begun listing ChatGPT as a co-author. OpenAI holds the opinion that text written by AI is not “wholly generated by a human or wholly generated by an AI” and that the individual must take responsibility for what the AI produces [14]. Ethical considerations are increasingly emphasized in scholarly publishing, with organizations such as COPE recommending transparent disclosure of AI assistance in academic work. Ultimately, the quality of the prompt given to an AI chatbot greatly affects the finished product. While this is something to consider, we may not see a definitive answer in terms of the “correct” way to approach AI policies in medicine since this technology is so new.

Despite the valid ethical concerns, one can make the case that ChatGPT and other generative models like it level the playing field. AI models could potentially be utilized because they could help those underrepresented in medicine, those who are first-generation in higher education, or those from lower socioeconomic statuses who may not have the time, connections, or resources to navigate the application process, and sometimes assist with editing or suggesting changes to their work where needed. AI language models like ChatGPT and Google Bard are free and easily accessible tools that could, in theory, be utilized to enhance an application. They can evaluate personal statements for grammar and syntax and improve the clarity and conciseness of whatever the writer inputs. Aside from picking out instances of plagiarism or AI-generated content, they could even be used as a tool to objectively critique personal statements and help prevent potential biases that may arise with human reviewers. Nonetheless, concerns remain about equity if AI access or proficiency varies across applicant groups. Moreover, while Stanford’s DetectGPT, Scribbr’s tool, and a similar tool used by Turnitin boast high rates of distinguishing AI from humans, the detection of AI-generated text is still not perfect. In the human-written statements, which were compiled before the advent of generative AI, one statement still returned an AI-generated content percentage of 20%. For this reason, while these AI detection tools can be helpful, their outputs should be taken with a healthy dose of skepticism, at least for the time being.

Another consideration and key limitation for this study is that the sample size of raters was small, and the observed lack of a significant difference in the categories of readability, originality, persuasiveness, and interview desirability may in fact be a significant one with a sufficient sample size of qualified reviewers and statistical power. Moreover, limited access to de-identified statements further constrained the number of essays that could be evaluated. This study included only 10 personal statements, resulting in an estimated post hoc power of 14.1% to detect a medium effect size. Although not part of the formal study protocol, one otolaryngologist and one lawyer independently attempted to identify whether each statement was AI-generated or human-written. The ENT was correct in nine out of the 10 essays, while the attorney was correct on 50% of the essays. While anecdotal, this observation may support the hypothesis that domain-specific familiarity with ERAS statements enhances intuitive recognition of AI-written content.

While the findings are valuable for raising awareness and guiding future research, the small sample size limits statistical confidence. Future studies should formally assess this detection ability, as well as the differences between AI- and human-written texts, in a larger and more systematic way. For residency programs looking to promote fielding authentic personal statements from applicants, potential strategies may include (1) incorporating AI-detection software into application screening; (2) designing structured prompts or essays that require applicant reflection beyond standard personal statements; and (3) developing policies to clarify acceptable vs. prohibited uses of AI in application materials.

## Conclusions

The integration of generative AI, like ChatGPT, among others, into the residency application process is a notable emerging factor in residency application evaluation that warrants further attention. AI-generated personal statements were rated comparably to applicant-written statements, underscoring the potential of generative AI to mimic human-authored application materials. While detection tools demonstrated strong performance in distinguishing AI content, they remain imperfect. These results highlight the importance of larger-scale studies and the need for residency programs to establish ethical guidelines and consistent practices for evaluating AI-assisted personal statements.

## Appendices

Appendix A: Grading rubric

**Table 2:** Personal Statement Grading Scale

Category	Criteria description	1 (Poor)	2 (Fair)	3 (Good)	4 (Very Good)	5 (Excellent)
Originality	The extent to which the personal statement presents unique insights, experiences, and creative expression.	Heavily Cliché	Somewhat Original	Moderately Original	Very Original	Exceptionally Original
Readability	How easily the personal statement can be read and understood, including structure, grammar, and flow.	Difficult to Read	Somewhat Readable	Moderately Readable	Very Readable	Exceptionally Readable
Convincing nature	The effectiveness of the statement in persuading the reader of the writer’s qualifications, passion, and suitability for the field of otolaryngology.	Not Convincing	Slightly Convincing	Moderately Convincing	Very Convincing	Exceptionally Convincing
Desire to meet writer	The degree to which the statement generates interest in the writer, making the reader eager to meet or learn more about them.	No Desire to Meet	Slight Desire to Meet	Moderate Desire to Meet	High Desire to Meet	Very High Desire to Meet

Appendix B: Prompt and output

**Table 3:** Prompt used and AI-generated output

AI essay	Prompts used in ChatGPT 4.0	Word output
1	“Write me a compelling personal statement for an Otolaryngology residency application that includes personal life experiences, like living with medical conditions or helping family with diseases. Feel free to include talking about other unique hobbies, tying them towards why I would be a great Otolaryngology residency candidate. Feel free to include made-up but meaningful and vividly detailed patient experiences, or detail research experiences related to Otolaryngology. Finally, say why I would be a good Otolaryngology resident and a good fit for any Otolaryngology program. Be sure to add specific and vivid details, names, and imagery to make the statement more engaging and believable. Write no less than 600 and no more than 850 words. Use this scale to guide you in producing a high quality statement:” (prompt included image of rubric)	596
2		541
3		645
4		596
5		614
